# CHALLENGES IN THE FIGHT AGAINST THE COVID-19 PANDEMIC IN UNIVERSITY HOSPITALS

**DOI:** 10.1590/1984-0462/2020/38/2020086

**Published:** 2020-04-22

**Authors:** Eduardo Alexandrino Servolo Medeiros

**Affiliations:** a Division of Infectious Diseases, Department of Medicine, Escola Paulista de Medicina, Universidade Federal de São Paulo, São Paulo, SP, Brazil.; b Hospital Infection Control Committee of Hospital São Paulo, São Paulo, SP, Brazil.

We are living the most important pandemic in recent world history, caused by a novel coronavirus (SARS-CoV-2), with a significant impact on the economy, public health, and mental health of the entire society. São Paulo is the epicenter of the epidemic in Brazil. Brazilian university hospitals - centers for professional training and qualification, as well as knowledge production - have a major role in combating this epidemic.

Coronaviruses belong to a large family of viruses and, for 60 years, have been a known cause of respiratory infection in humans and animals. In December 2019, a novel coronavirus was identified as responsible for the flu syndrome and severe pulmonary complications, the COVID-19. Its origin, still uncertain, is probably related to a mutation in the coronavirus that infects bats, breaking the genetic barrier to adapt to a new species. The original site of transmission was a seafood and live animal market in the city of Wuhan, China. The first cases were linked to individuals who frequented this market. Later, the virus infected family members and, in geometrical progression, nearby provinces, expanding to several countries in all continents.^1^ ^,^ ^2^

The virus is highly contagious through droplets and contact. Estimates indicate that an infected person can transmit the virus to two to four people.^1^ The angiotensin-converting enzyme 2 (ACE2), found in the lower respiratory tract of humans, has been identified as a cellular receptor for SARS-CoV-2 and plays an important role in the pathogenesis and spread of the virus. The S-glycoprotein on the surface of the coronavirus can bind to the ACE2 receptor on the surface of cells, especially lung cells, rich in ACE2 receptors. The ribonucleic acid (RNA) of the viral genome is released inside the cell, starting the encoding of structural and accessory proteins, with subsequent release of new viruses. This process results in the release of cytokines with intense inflammatory response, leading to respiratory failure, shock, and thromboembolic phenomena related to disseminated intravascular coagulation.^3^ ^,^ ^4^

Children probably develop milder and oligosymptomatic clinical presentations because the maturity and binding ability of ACE2 might be lower in this population than in adults. This is concerning from an epidemiological perspective, since children can be important reservoirs, becoming sources of infection.^4^

The incubation period is, on average, five days, ranging from two to 14 days. Most adults or children infected with SARS-CoV-2 present flu syndrome (90%) with mild symptoms, but some individuals, especially older adults and those with comorbidities, such as cardiovascular or lung diseases, diabetes, and hypertension, might progress to severe conditions: respiratory failure, multiple organ failure, and death. The fatality rate is 2 to 5%.^2^ Although children can acquire the infection, they usually have a good prognosis and rarely present complications.^4^

No country is prepared to face the COVID-19 epidemic, which imposes substantial negative impacts on the economy, medical care, and mental health of the society as a whole. Brazil, particularly São Paulo, which declared a state of emergency, has implemented adequate preventive measures in accordance with the epidemiological scenario.^5^

Rigorous social distancing of the population is crucial, in addition to educational campaigns for hygiene and the proper use of masks. Social distancing measures should be constantly evaluated because if they are lifted before the appropriate moment, that is, before the end of community transmission, we will have a new wave and growth in cases of infection. The suspension of academic calendars, including schools of Medicine, with revision of college admission exams and evaluations of medical residency, will have a significant impact.

The main challenges for hospitals, especially university hospitals, are: reorganize the care provided, increase the number of intensive care unit beds, guarantee the availability of personal protective equipment, particularly protection masks and gowns, which are in short supply, and have enough tests for diagnosis. Many health professionals are getting sick and having to stay out of work, which can lead to the collapse of hospital care, as seen in countries such as Italy and Spain. Research support is essential in the search for effective medications - in the clinical protocol phase at the moment - and a vaccine, which will probably be available only in 2021, after this pandemic is over.

As health professionals, we must prepare for the worse in the coming weeks, protect ourselves, have hope, and be on the front line, contributing to this critical and historic fight against this novel coronavirus.

## References

[1] Benvenuto D, Giovannetti M, Ciccozzi A, Spoto S, Angeletti S (2020). The 2019-new coronavirus epidemic: evidence for virus evolution. J Med Virol.

[2] Zhu N, Zhang D, Wang W, Li X, Yang B (2020). A novel coronavirus from patients with pneumonia in China, 2019. N Engl J Med.

[3] Chan JF, Yuan S, Kok KH, Kai-Wang To K, Chu H (2020). A familial cluster of pneumonia associated with the 2019 novel coronavirus indicating person-to-person transmission: a study of a family cluster. Lancet.

[4] Dong Y, Mo X, Hu Y, Qi X, Jiang F (2020). Epidemiological characteristics of 2143 pediatric patients with 2019 coronavirus disease in China. Pediatrics.

[5] Brazil - Diário Oficial de São Paulo Decreto Nº 64.879 que reconhece o estado de Calamidade Pública no estado por conta da pandemia do Coronavírus (COVID-19) 21 de Março de 2020.

